# The Effects of Empagliflozin, an SGLT2 Inhibitor, on Pancreatic β-Cell Mass and Glucose Homeostasis in Type 1 Diabetes

**DOI:** 10.1371/journal.pone.0147391

**Published:** 2016-01-25

**Authors:** Sam Tsz Wai Cheng, Lihua Chen, Stephen Yu Ting Li, Eric Mayoux, Po Sing Leung

**Affiliations:** 1 School of Biomedical Sciences, The Chinese University of Hong Kong, Sha Tin, Hong Kong; 2 Boehringer-Ingelheim Pharma GmbH & Co KG, Biberach, Germany; Universidad Miguel Hernández de Elche, SPAIN

## Abstract

The novel sodium glucose co-transporter 2 (SGLT2) inhibitor empagliflozin has recently been reported to improve glycemic control in streptozotocin-induced type 1 diabetic rats in an insulin-independent manner, via an increase in urinary glucose output. We investigated the potential of empagliflozin to recover insulin pathways in type 1 diabetes by improving pancreatic β-cell mass. Blood glucose homeostasis was assessed by an intraperitoneal glucose tolerance test. Serum insulin levels and insulin mRNA expression were determined using commercial insulin ELISA kits and real-time quantitative polymerase chain reaction, respectively. Immunohistochemistry was used to investigate β-cell areas, β-cell proliferation, apoptosis of pancreatic β-cells, and reactive oxygen species production in the pancreatic β-cells. Results showed that glucose tolerance was significantly improved in streptozotocin-induced type 1 diabetic mice treated with empagliflozin. Empagliflozin-treated mice also showed an increase in insulin mRNA expression. Higher serum insulin levels were detected in mice treated with empagliflozin compared with the vehicle group. Immunohistochemistry indicated that β-cell area/total pancreatic area and the expression of cell proliferation marker Ki-67 (co-stained with insulin) were significantly enhanced by empagliflozin treatment. These effects were due, probably, to a reduction in apoptosis and reactive oxygen species in the pancreatic β-cells. Taken together, the results of this study indicate that empagliflozin may have a beneficial effect on preserving β-cell regeneration, thus improving blood glucose homeostasis in type 1 diabetes mellitus, probably via the protection of pancreatic β-cell from glucotoxicity-induced oxidative stress.

## Introduction

Type 1 diabetes mellitus (T1DM), also known as insulin-dependent diabetes mellitus or juvenile onset diabetes, represents 5–10% of all diagnosed cases of diabetes in the United States [1]. T1DM is caused by autoimmune pancreatic β-cell destruction with consequent severe insulin deficiency [2]. T1DM is currently incurable and patients rely on daily insulin injections. However, the use of exogenous insulin injections to control blood glucose level puts patients with T1DM at risk of hypoglycemia [3]. In view of this, there is a great interest in the development of non-insulin-mediated anti-hyperglycemic therapies for the management of T1DM.

In healthy humans, the kidneys filter 160 to 180 grams of glucose per day, which is subsequently reabsorbed into the bloodstream via the renal proximal convoluted tubule [4]. Studies in knockout mice indicate that, in euglycemia, the sodium glucose co-transporter 2 (SGLT2) and the sodium glucose co-transporter 1 (SGLT1) are responsible for the reabsorption of 97% and 3% of filtered glucose, respectively [5]. In studies in proximal tubular epithelial cells from patients with type 2 diabetes mellitus (T2DM) and in diabetic rodent models, SGLT2 expression and renal glucose reabsorption were increased [6–7]. Basic scientific research on SGLT2 deletion has also shown protective effects on pancreatic islets in animal models of T2DM [8]; in those study, the pancreatic β-cells of SGLT2 knockout mice fed with a high-fat diet were protected from hyperglycemia and glucose intolerance, which was explained by a 3-fold increase in urine output and a 500-fold increase in glucosuria due to SGLT2 deletion [8].

In light of these findings, there is a rationale to develop drugs to pharmacologically block the action of renal SGLT2 for the management of diabetes. Phlorizin has been shown to effectively improve glucose tolerance and insulin resistance in diabetic rodents, as illustrated by reduction of blood glucose and HbA1c levels [9]. However, due to its relatively low specificity for SGLT2, phlorizin also blocks intestinal sodium glucose co-transporter 1, leading to unacceptable intestinal side effects such as diarrhea [4]. This led to the development of more selective and potent SGLT2 inhibitors that are suitable for treating patients with diabetes [10].

The development of specific SGLT2 inhibitors provides hope to both patients with T1DM and T2DM, due to their insulin-independent mechanism of action, i.e. increased urinary glucose excretion. SGLT2 inhibitors have been shown to consistently improve glycemic control and pancreatic islets function, as well as preserving islet morphology in T2DM [4, 11–12]. Empagliflozin (BI 10773), a novel and selective SGLT2 inhibitor, was recently approved for the treatment of T2DM. Although the amount of reabsorbed glucose increases up to two- to three-fold during hyperglycemia, this novel SGLT2 inhibitor can significantly reduce HbA1c, fasting blood glucose levels, body weight, and total daily insulin dosing without increasing the risk of hypoglycemia; it may also confer renal protection by minimizing the glomerular hyperfiltration that arises in diabetes [11–13].

Although SGLT2 inhibitors are recommended for treating patients with T2DM, studies are currently being undertaken to explore their potential for the treatment of T1DM [14–15]. A recent study documented the anti-hyperglycemic effect of empagliflozin in a streptozotocin (STZ)-induced T1DM animal model [14]. Moreover, dapagliflozin increased urinary glucose excretion in adult patients with T1DM; however, larger randomized, controlled studies of SGLT2 inhibitors are required [16]. The present study was, therefore, designed to investigate into the effects of empagliflozin on islet β-cell mass and function.

## Materials and Methods

### Animal model and experimental design

Male C57BL/6J mice (10 weeks of age) were obtained from the Laboratory Animal Services Center of the Chinese University of Hong Kong. Mice were housed under a 12 hour light/dark cycle (7am-7pm) in temperature and humidity controlled rooms and were provided with *ad libitum* rodent chow (Teklad 7001, 4.4%; Harlan Teklad Global Diets) and water. T1DM was induced by intraperitoneal injection of streptozotocin (Sigma-Aldrich, USA) at 50mg/kg for 5 consecutive days. Non-fasting blood glucose levels were measured for three times in three separate days following the last injection of streptozotocin. T1DM was considered successfully induced in mice with an average blood glucose level greater than 16mM and these mice were selected for subsequent drug administration and experiments.

Empagliflozin was dissolved in hydroxy ethyl cellulose (HEC) and administered to mice in the experimental group (3 or 10 mg/kg) by oral gavage once daily for 8 days, whereas the vehicle group was given same volume of HEC alone. The experimental procedures were approved by the Animal Experimentation Ethics Committee of the Chinese University of Hong Kong (reference number: 12/062/MIS-5).

### In vivo glucose homeostasis

Blood glucose levels were measured in blood drawn from the tail vein, using a glucometer (Bayer Corporation, USA). An intraperitoneal glucose tolerance test was performed 24 hours after the last dose of treatment. Briefly, mice were challenged with glucose (1 g/kg) after a 6-hour fast, and blood glucose was measured 0, 15, 30, 60, 90, and 120 minutes thereafter. Area under the curve (AUC) of glucose was then calculated. Serum was collected from blood withdrawn from heart after sacrifice. Serum insulin content was determined using commercial ultra-high mouse insulin ELISA kits (Antibody and Immunoassay Services, HKU).

### Immunohistochemistry

Pancreata were rinsed in phosphate-buffered saline (PBS) to remove any blood retained on the surface and the tissues were embedded in O.C.T. compound (Sakura, Japan) and frozen. Cryostat sections were cut (6 μm thick) and mounted on glass slides. Sections were then blocked in 2% bovine serum albumin (Sigma-Aldrich, USA) with 0.1% Triton X-100 (Sigma-Aldrich, USA) for 1 hour at 25°C.

For the assessment of α-cell and β-cell areas, slides were incubated overnight at 4°C with rabbit polyclonal anti-insulin antibody (1:400, Santa Cruz, USA) and mouse monoclonal anti-glucagon antibody (1:250, Abcam, USA), and then incubated for 2 hours at 25°C with Alexa Fluor^®^ 568 donkey anti-rabbit antibody (1:2000, Life Technologies, USA) and Alexa Fluor^®^ 488 goat anti-mouse antibody (1:2000, Life Technologies, USA). For the measurement of β-cell proliferation, slides were incubated overnight at 4°C with guinea pig polyclonal anti-insulin antibody (1:400, Life Technologies, USA) and rabbit polyclonal anti-Ki67 antibody (1:250, Abcam, USA), and then incubated for 2 hours at 25°C with Alexa Fluor^®^ 488 goat anti-guinea pig antibody (1:2000, Life Technologies, USA) and Alexa Fluor^®^ 568 donkey anti-rabbit antibody (1:2000, Life Technologies, USA). For the detection apoptosis of pancreatic β-cells, an *in situ* cell death detection kit (Roche, Swiss) was used. Slides were incubated with citrate buffer (Thermo, USA) at 58°C for 35 minutes and then at 25°C for 20 minutes to achieve heat-induced epitope retrieval. Slides were then incubated overnight at 4°C with terminal deoxynucleotidyl transferase-mediated dUTP nick-end labeling (TUNEL) reaction mixture. After that, slides were washed 3 times with PBS, for 10 minutes each time, then incubated with rabbit polyclonal anti-insulin antibody (1:400, Santa Cruz, USA) for 4 hours and then incubated for 2 hours at 25°C with Alexa Fluor^®^ 568 donkey anti-rabbit antibody (1:2000, Life Technologies, USA). For the evaluation of reactive oxygen species (ROS) production in pancreatic β-cells, slides were incubated overnight at 4°C with guinea pig polyclonal anti-insulin antibody (1:400, Life Technologies, USA) and then incubated for 2 hours at 25°C with Alexa Fluor^®^ 488 goat anti-guinea pig antibody (1:2000, Life Technologies, USA). Dihydroethidium (5 μM) was dissolved in dimethyl sulfoxide (Sigma-Aldrich, USA) and subsequently added to the pancreatic sections and incubated at 37°C for 30 minutes.

Slides were incubated with the nuclear counterstain 4′6′-diamidino-2-phenylindole (DAPI) (1:5000, Life Technologies, USA) for 10 minutes to label the nuclei of cells. The slides were then washed 3 times with PBS, for 10 minutes each time. The slides were then mounted with VectaShield^®^ mounting medium to preserve fluorescence, and observed under a fluorescent microscope. All digital images were acquired by a fluorescence microscope equipped with a DC 200 digital camera (Leica Microsystems, Germany) and then analyzed by Leica Qwin image analysis software (Leica, Germany).

### Real-time polymerase chain reaction

Trizol reagent (Invitrogen, Waltham, MA, USA) was used for total RNA extraction from fresh pancreata according to the manufacturer's instructions. Real-time quantitative polymerase chain reaction was performed using SYBR Green (Life Technologies, USA) labeling and specific primers: Mouse Gapdh: (F) 5’-GCA CAG TCA AGG CCG AGA AT-3’ (R) 5’-GCC TTC TCC ATG GTG GTG AA-3’ and Mouse Insulin: (F) 5’-AGC GTG GCT TCT TCT ACA CAC-3’ (R) 5’-CTG GTG CAG CAC TGA TCT ACA-3’. Relative expression was normalized as a percentage of Gapdh and calculated using the comparative threshold cycle method (2^−ΔΔCT^).

### Statistical data analysis

Data are shown as means±S.E.M. Between-group differences were analyzed by two-tailed Student t-test or one-way ANOVA, followed by Tukey *post hoc* test, in which p<0.05 was considered statistically significant.

## Results

### Empagliflozin improved glucose tolerance in T1DM mice

**Fig 1:**
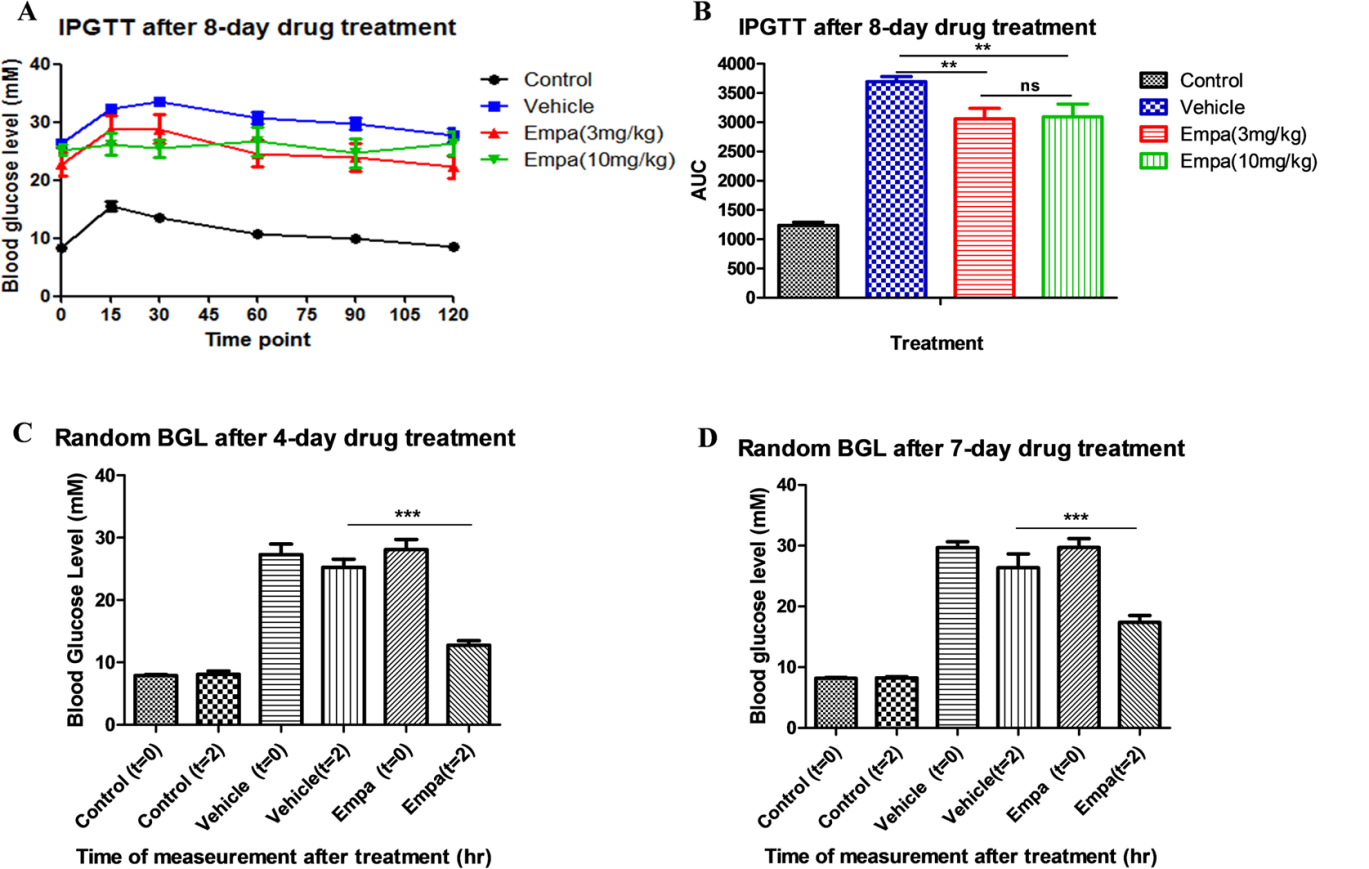
Effects of empagliflozin on glucose homeostasis. (A) Blood glucose levels in vehicle- and empagliflozin-treated (3mg/kg for 8 days) mice during an intraperitoneal glucose tolerance test. (B) Total change in blood glucose levels over time, expressed as area under curve (AUC). (C) The effect of empagliflozin on random blood glucose levels (BGL) in T1DM mice after (A) 4-day and (D) 7-day drug administration (3mg/kg). Data are expressed as means±S.E.M. *P <0.05; **P < 0.01 vs vehicle group. (*n* = 9–11 for each group).

T1DM mice treated with HEC (vehicle group) demonstrated glucose intolerance after challenge by intraperitoneal injection of glucose compared with normal mice (Fig 1A and 1B; AUC normal mice 1237±55 vs vehicle 3694±86). Interestingly, glucose intolerance was significantly improved after 8 days of empagliflozin treatment at either dose (Fig 1A; 3mg/kg empagliflozin 3058±180 vs 10mg/kg empagliflozin 3090±219). Therefore, acute treatment with empagliflozin had a beneficial effect on hyperglycemia and glucose intolerance. Since there were no significant differences in blood glucose homeostasis with the two different doses of empagliflozin, and random blood glucose levels of T1DM mice were significantly improved by 3mg/kg of empagliflozin (Fig 1C and 1D), we investigated the effect of the lower dose of empagliflozin (3mg/kg) on preserving β-cell mass and function.

### Pancreatic insulin mRNA and circulating levels of insulin were elevated in T1DM mice treated with empagliflozin

**Fig 2:**
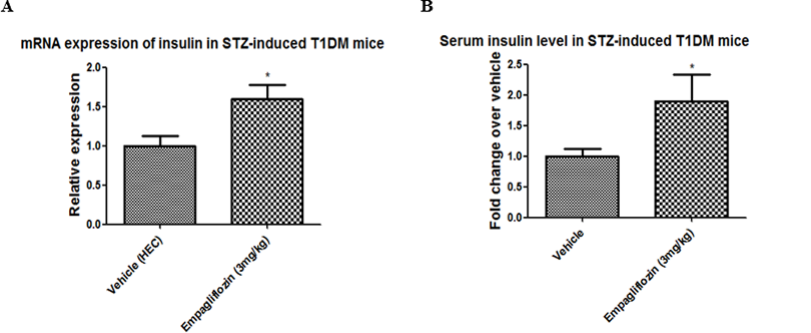
Effects of empagliflozin on insulin expression and serum levels. The effect of vehicle or empagliflozin treatment (3mg/kg) for 8 days on insulin expression (A) mRNA, and (B) serum levels. Data are expressed as mean±S.E.M. *P <0.05 vs vehicle group. (*n* = 10–11 for each group for serum insulin; *n* = 8 for each group for mRNA).

To determine the influence of empagliflozin on insulin mRNA levels in pancreata and serum insulin levels in plasma, real-time polymerase chain reaction and commercial insulin ELISA kit were used, respectively. Mean pancreatic insulin mRNA levels in T1DM mice treated with empagliflozin (3mg/kg) were significantly increased by 60% compared with the vehicle group (Fig 2A; vehicle 1.00±0.13 vs empagliflozin 1.60±0.18). Similar results were obtained for circulating insulin levels: serum insulin levels were significantly elevated by 90% in T1DM mice treated with empagliflozin (3mg/kg) compared with the vehicle group (Fig 2B; vehicle 1.00±0.12 vs empagliflozin 1.90±0.43).

### Pancreatic β-cell mass in T1DM mice was enhanced by empagliflozin treatment

**Fig 3:**
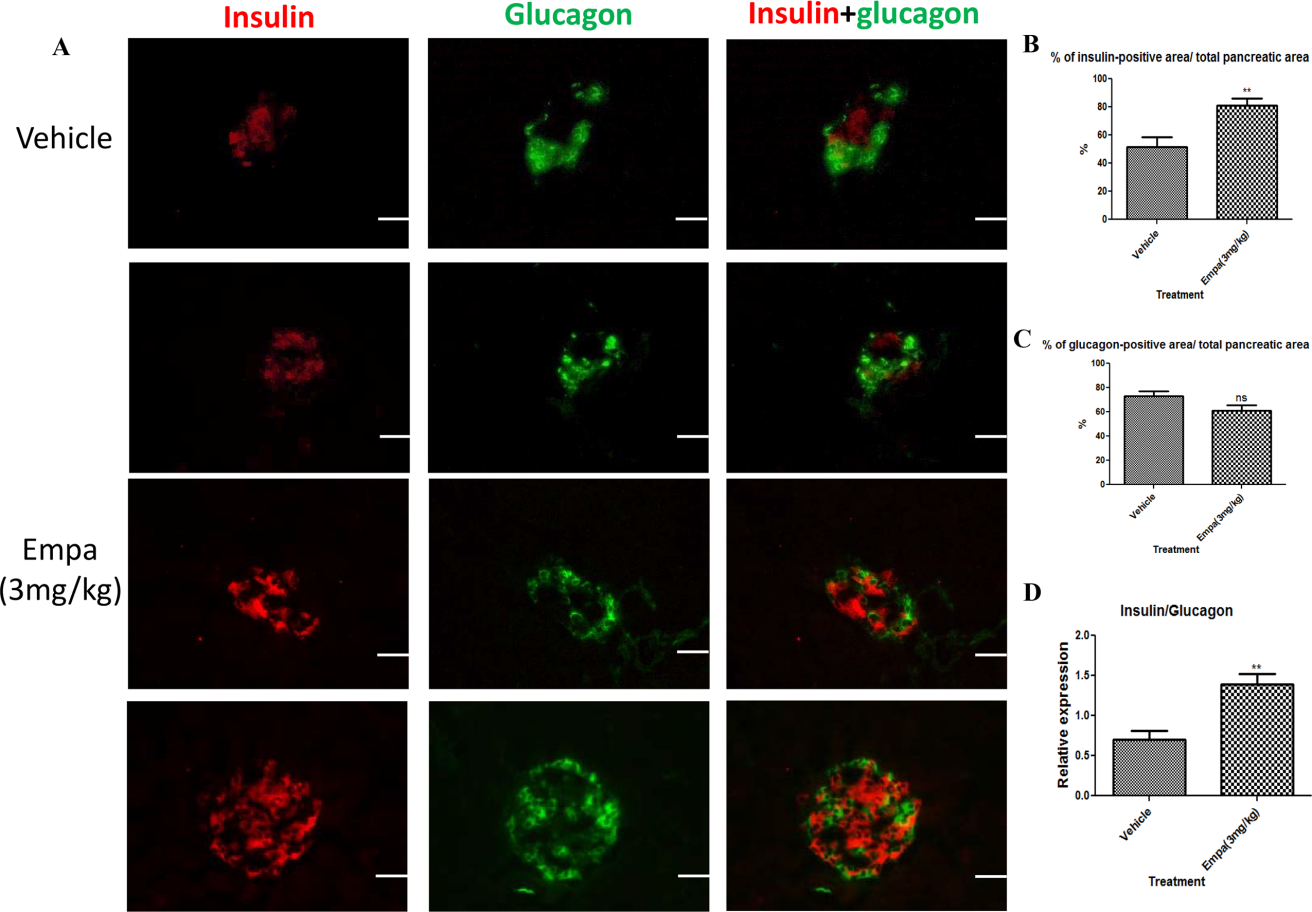
Assessment of insulin and glucagon producing cells after empagliflozin treatment. β-cell mass was examined from the whole pancreata isolated from T1DM mice after treatment with empagliflozin (3mg/kg) or vehicle for 8 days. (A) Double fluorescence labeling using glucagon (green) and insulin (red) in pancreas sections from vehicle- and empagliflozin-treated mice. (B) Bar graph shows the % of β-cell area/ total pancreatic area. (C) Bar graph shows the % of α-cell area/ total pancreatic area. (D) Bar graphs show the mean area of insulin/glucagon ratio per pancreatic cross-section. Data are expressed as mean±S.E.M. Scale bar = 100 μm. **P<0.01 vs vehicle group. (*n* = 10–12 for each group).

To determine the effect of empagliflozin on β-cell mass in pancreatic islets immunohistochemical staining with insulin and glucagon was applied to pancreatic cryosections. (Fig 3A) After treatment with empagliflozin (3 mg/kg) for 8 days, the % of β-cell area/ total pancreatic area significantly increased. (Fig 3B; vehicle 51.35±7.06 vs empagliflozin 81.09±4.77). Although the % of α-cell area/ total pancreatic area has a trend to decrease, it was not statistically significant. (Fig 3C; vehicle 73.22±3.99 vs empagliflozin 61.18±4.50). Moreover, the β-cell to α-cell ratio in T1DM mice was increased compared with the vehicle group (Fig 3D; vehicle 0.70±0.11 vs empagliflozin 1.39±0.13)

### β-cell proliferation in T1DM mice was increased by empagliflozin treatment

**Fig 4:**
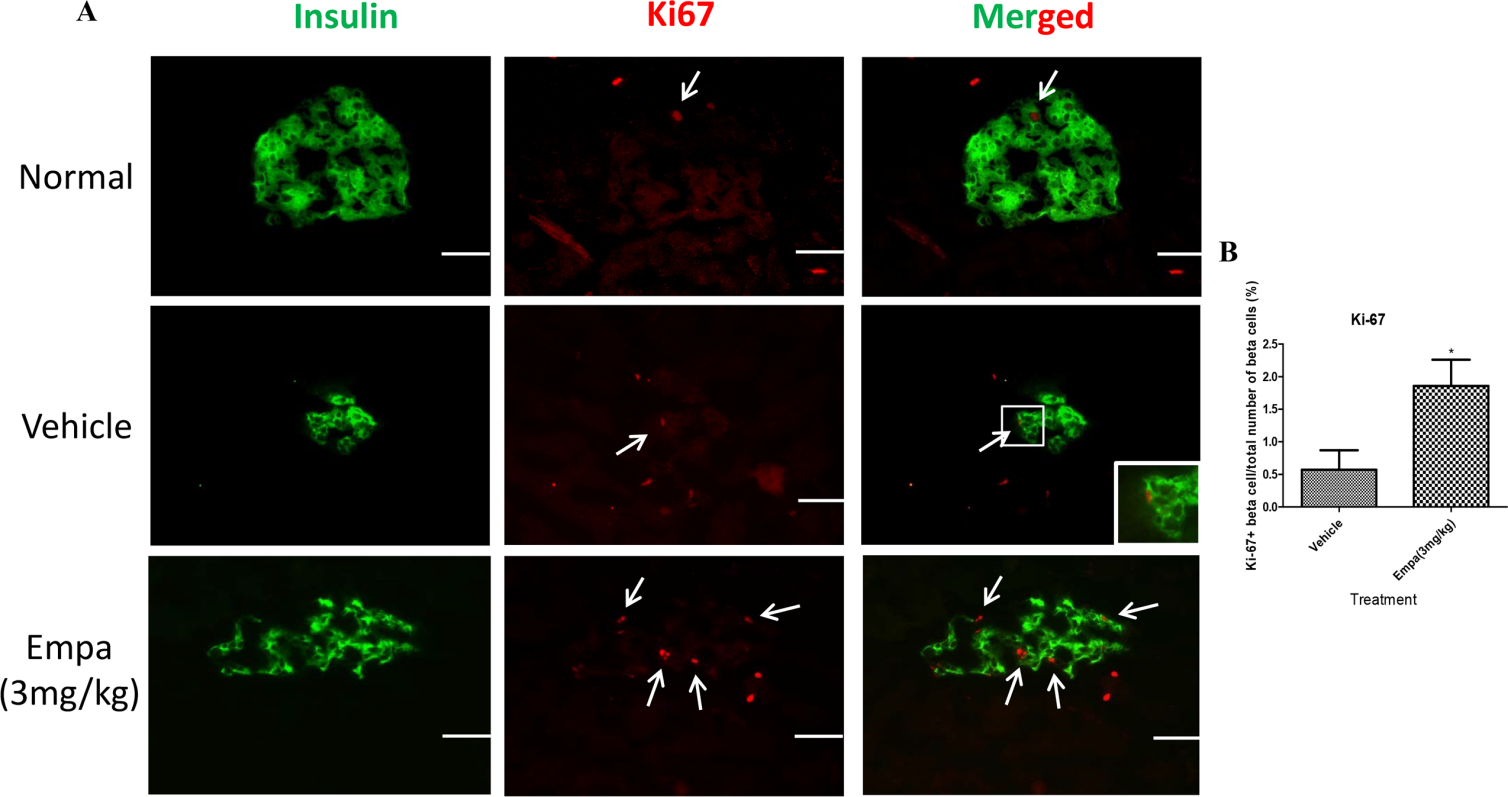
Measurement of β-cell proliferation after empagliflozin treatment. Immunohistochemical staining of Ki-67 of whole pancreata was performed from T1DM mice after treatment with empagliflozin (3mg/kg) or vehicle for 8 days. (A) Double fluorescence labeling using insulin (green) and Ki-67 (red) in pancreatic sections from vehicle- and empagliflozin-treated mice. Arrows indicate Ki-67-positive β-cells. (B) Bar graphs show the % of Ki-67 positive β-cell/ total number of β-cells. Data are expressed as mean±S.E.M. Scale bar = 100 μm. *P<0.05 vs vehicle group. (*n* = 8 for each group).

β-cell proliferation was assessed by counting Ki-67/insulin/DAPI-copositive nuclei, which represent proliferating β-cells in each pancreatic islet. The number of the Ki-67-positive cells to total β-cells in the empagliflozin-treated group (3 mg/kg) was greater than that in the vehicle group (Fig 4; vehicle 0.57±0.30 vs empagliflozin 1.86±0.40).

### Apoptosis in the pancreatic β-cells of T1DM mice was reduced by empagliflozin treatment

**Fig 5:**
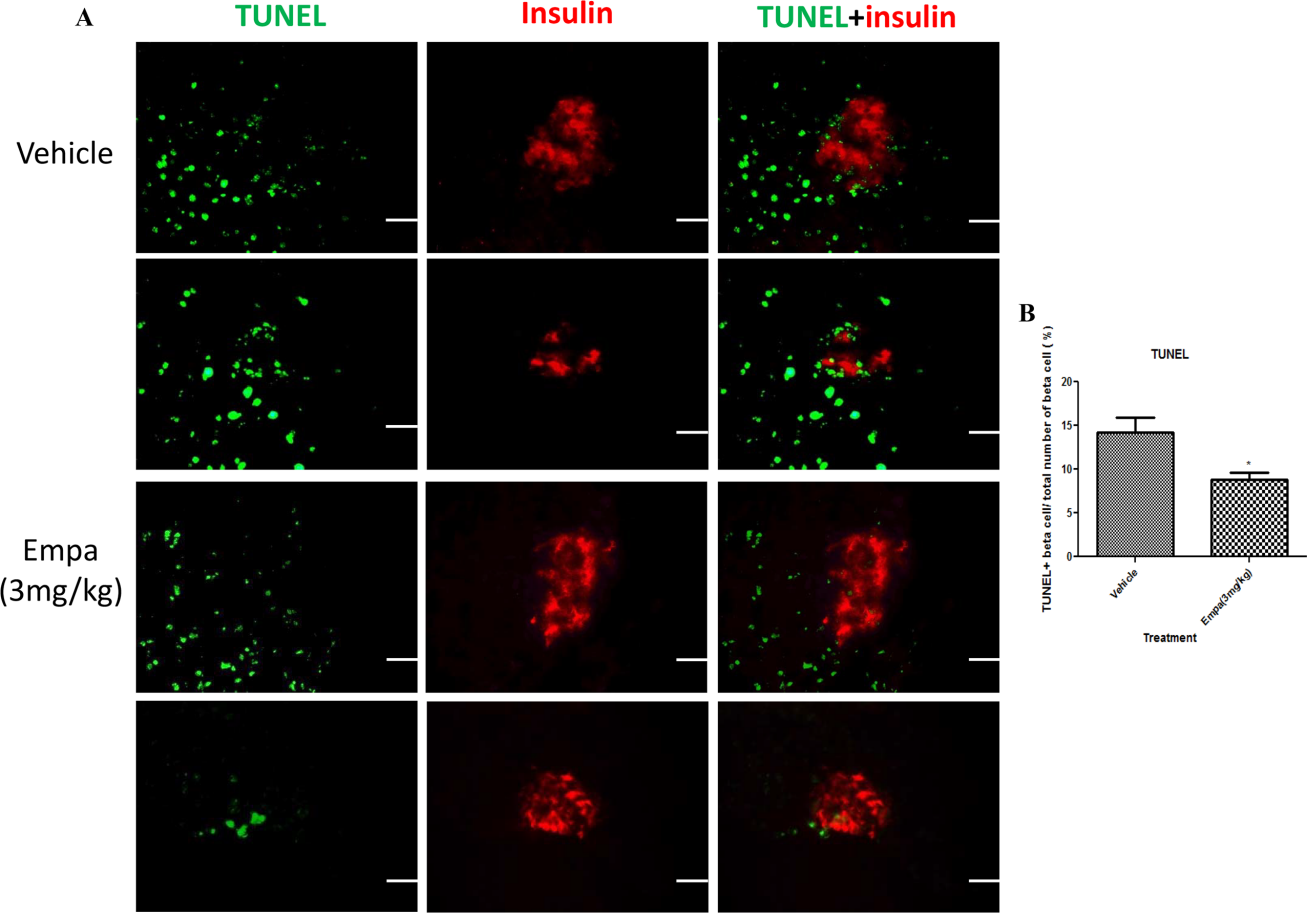
Measurement of β-cell apoptosis after empagliflozin treatment. TUNEL assay of whole pancreata was performed from T1DM mice after treatment with empagliflozin (3mg/kg) or vehicle for 8 days. (A) Double fluorescence labeling using insulin and terminal deoxynucleotidyl transferase-mediated dUTP nick-end labeling (TUNEL) assay in pancreas sections from vehicle- and empagliflozin-treated mice. (B) Bar graphs show the % of TUNEL positive β-cell/total number of β-cells. Data are expressed as mean±S.E.M. Scale bar = 100 μm.*P<0.05 vs vehicle group. (*n* = 10–12 for each group).

We quantified the level of apoptosis of pancreatic β-cells using immunofluorescence labeling TUNEL assay in T1DM mice treated with empagliflozin (3 mg/kg) or vehicle. The overlap of DAPI labeling, insulin and TUNEL labeling was scored as apoptotic pancreatic β-cells. TUNEL-positive β-cells per pancreatic islet were significantly decreased in empagliflozin-treated mice compared with the vehicle group (Fig 5; vehicle 14.26±1.71 vs empagliflozin 8.79±0.86).

### Pancreatic β-cell ROS level was decreased by empagliflozin treatment

**Fig 6:**
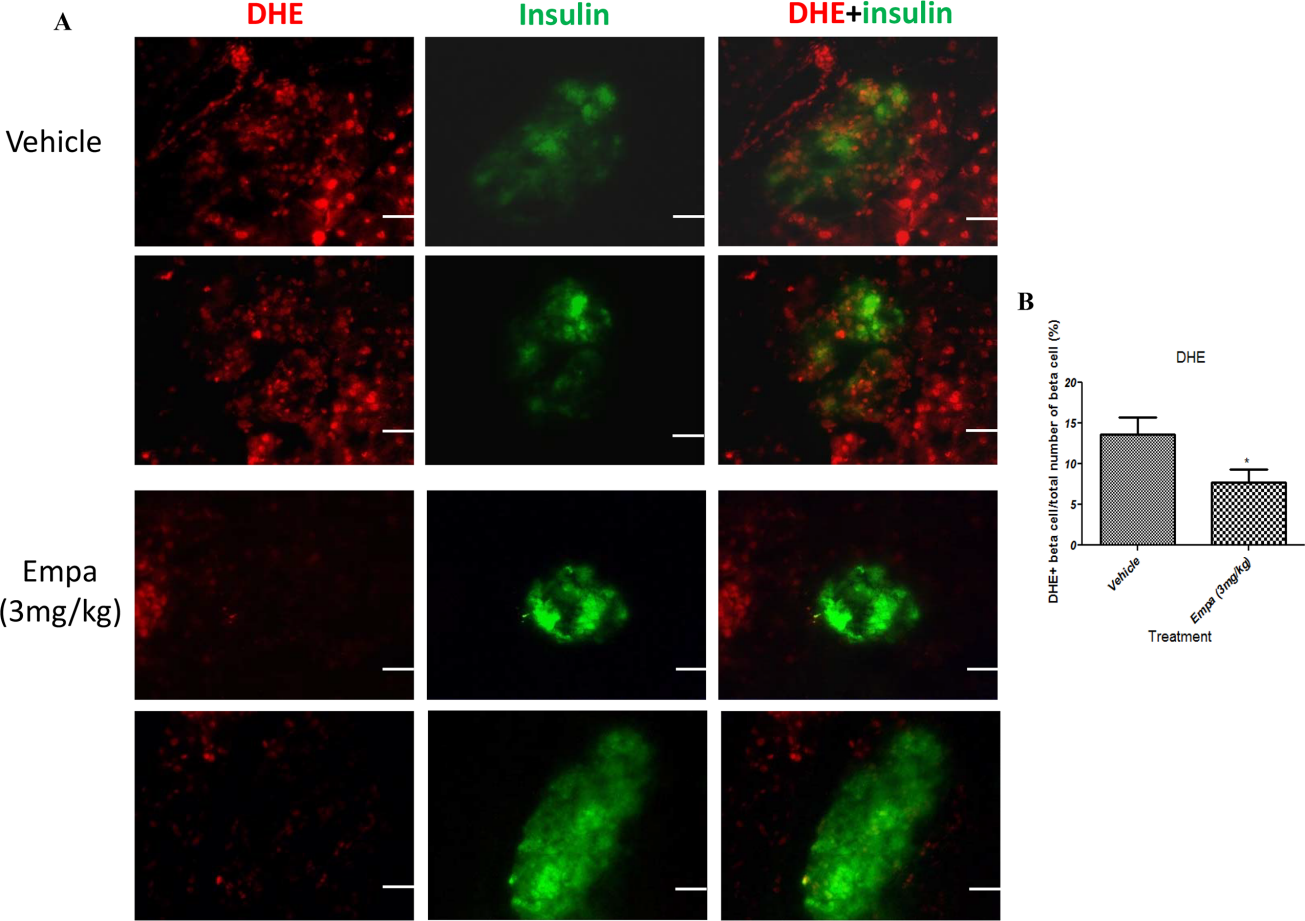
Measurement of β-cell ROS levels after empagliflozin treatment. DHE staining of whole pancreata was performed from T1DM mice after treatment with empagliflozin (3mg/kg) or vehicle for 8 days. (A) Double fluorescence labeling using insulin and dihydroethidium (DHE) staining in pancreas sections from vehicle- and empagliflozin-treated mice. (B) Bar graphs show the % of DHE positive β-cell/total number of β-cells. Data are expressed as mean±S.E.M. Scale bar = 100 μm. *P<0.05 vs vehicle group. (*n* = 10–12 for each group).

To determine the influence of empagliflozin (3 mg/kg) on ROS levels in the pancreatic β-cells, *in situ* staining with dihydroethidium was applied to pancreatic cryosections. Analysis of % of DHE positive β-cell/ total number of β-cells revealed a significant decrease in superoxide formation in empagliflozin-treated T1DM mice compared with the vehicle group (Fig 6; vehicle 13.64±2.09 vs empagliflozin 7.75±1.54).

## Discussion

T1DM is initiated by autoimmune destruction of pancreatic β-cells in patients with genetically predisposed impaired immunity [17]. In addition to identifying defects in islet autoimmunity in patients with T1DM, novel oral hypoglycemic drugs might enable the restoration of islet function. Of great interest in this context is the highly specific inhibition of the glucose transporter, SGLT2. As SGLT2 is responsible for mediating the majority of glucose reabsorption in the kidneys, biopharmaceutical companies have focused on the development of selective SGLT2 inhibitors. Several SGLT2 inhibitors have been approved for the clinical use to treat patients with T2DM [18–20]. Among them, empagliflozin is a member of this new class of oral anti-diabetic drugs, and its pharmacological mechanism-of-action is well established i.e. minimizing renal glucose reabsorption. SGLT2 inhibitors have been shown to improve insulin sensitivity, reverse glucotoxicity, and normalize glucose homeostasis in T2DM [11–12]. One of the major patient benefits of SGLT2 inhibition is that blood glucose levels are reduced without increasing the risk of hypoglycemia or producing weight gain, both of which are major and unpreventable side effects of other classes of oral anti-hyperglycemic drugs.

Patients with T1DM display a significant increase in maximal renal glucose reabsorption, making it difficult for them to control their blood glucose levels [21]. Interestingly, empagliflozin effectively lowered blood glucose levels in T1DM animal model and human subjects, by blocking the reabsorption of glucose in the proximal renal tubule [14–15, 22]. Renal gluconeogenesis is enhanced in the fasting and postprandial states in patients with diabetes [23–24]. This is due to up-regulation of phosphoenolpyruvate carboxykinase, a key regulator of renal gluconeogenes in the kidneys of diabetic rats and humans; in this regard, empagliflozin lowered blood glucose levels in Akita diabetic mice by attenuating the up-regulation of phosphoenolpyruvate carboxykinase [13].

The present study data have demonstrated, for the first time, the protective role of empagliflozin in the preservation of β-cell mass in T1DM. Our results obtained showed that treatment of T1DM mice with empagliflozin (3 or 10mg/kg) for 8 days significantly improved glucose tolerance. This beneficial effect can be explained by the increased urinary glucose output from the kidneys associated with empagliflozin, thus reducing plasma glucose levels. This observation is further supported by the results of a previous study in STZ-induced T1DM rats in which empagliflozin alone and in combination with insulin improved glycemic control. Indeed, when empagliflozin was added to insulin in this study, similar levels of glycemic control were achieved with lower doses of insulin and lower risk of hypoglycemia [14]. Interestingly, both mRNA and serum insulin levels in T1DM mice were significantly enhanced after 8-day treatment with empagliflozin, which might be due to the preservation of β-cell mass, as evidenced by the reduction of β-cell apoptosis as well as the stimulation of β-cell proliferation in the present study.

Accumulation of excess ROS in the body leads to oxidative stress, thus deleteriously affecting cell function. It is noteworthy that pancreatic islets are highly susceptible to oxidative stress, due to their low levels of intrinsic antioxidant systems [25]. Indeed, excessive ROS generation is one of the proposed mechanisms for glucotoxicity observed in diabetes. Chronic exposure of pancreatic β-cells to high concentrations of glucose causes suppression of insulin gene expression, as well as significant reductions in insulin content and secretion [26]. In the present study, empagliflozin significantly reduced the level of oxidative stress, which is believed to be induced by high blood glucose levels in T1DM. The maintenance of β-cell area and function is critical for the control of blood glucose levels within a narrow physiological range [27]; in corroboration with this observation, the present data showed an improvement in the maintenance of β-cell area and function after treatment with empagliflozin. This improvement was due, in part, to the increase in β-cell proliferation and the decrease in β-cell apoptosis. Previous studies indicated that SGLT2 expression in mouse and human is highly kidney-specific [28–29]. Interestingly, Bonner C et al demonstrated that SGLT2 expresses in human pancreatic α-cells and another SGLT2 inhibitor dapagliflozin triggers glucagon secretion in islets from patients [30]. However, we did not observe empagliflozin affecting neither glucagon expression (Fig 3C) nor secretion (data not shown) in T1DM mice. It may be due to differences in experimental models. It is plausible to propose that empagliflozin might not have a direct effect on β-cell proliferation, but the role of empagliflozin or SGLT2 in the regulation of pancreatic β-cell function and cell mass warrants further investigations.

Collectively, our data have shown, for the first time, that empagliflozin, a SGLT2 inhibitor, improves islet function and β-cell mass in pancreatic islets, likely by the reduction of oxidative stress within β-cell that translates to β-cell protection and higher insulin secretion. If endogenous insulin production is preserved in patients with T1DM, there is a possibility that they can reduce their reliance on exogenous insulin injections [31]. However, more clinical data are needed to verify the safety and efficacy of this class of drugs in treating patients with T1DM, particularly in patients with recurrent urinary tract infections and impaired renal function, respectively.
